# Dystonia genes functionally converge in specific neurons and share neurobiology with psychiatric disorders

**DOI:** 10.1093/brain/awaa217

**Published:** 2020-08-21

**Authors:** Niccolò E Mencacci, Regina H Reynolds, Sonia Garcia Ruiz, Jana Vandrovcova, Paola Forabosco, Alvaro Sánchez-Ferrer, Viola Volpato, UK Brain Expression Consortium, Juan A Botía, Karishma D'Sa, Paola Forabosco, Sebastian Guelfi, John Hardy, Jana Vandrovcova, Chris-Ann Mackenzie, Adaikalavan Ramasamy, Mina Ryten, Colin Smith, Daniah Trabzuni, Michael E Weale, International Parkinson’s Disease Genomics Consortium, Alastair J Noyce, Rauan Kaiyrzhanov, Ben Middlehurst, Demis A Kia, Manuela Tan, Henry Houlden, Huw R Morris, Helene Plun-Favreau, Peter Holmans, John Hardy, Daniah Trabzuni, Jose Bras, John Quinn, Kin Y Mok, Kerri J Kinghorn, Kimberley Billingsley, Nicholas W Wood, Patrick Lewis, Rita Guerreiro, Ruth Lovering, Lea R’Bibo, Claudia Manzoni, Mie Rizig, Mina Ryten, Sebastian Guelfi, Valentina Escott-Price, Viorica Chelban, Thomas Foltynie, Nigel Williams, Chingiz Shashakin, Nazira Zharkinbekova, Elena Zholdybayeva, Akbota Aitkulova, Kirsten Harvey, Michael E Weale, Kailash P Bhatia, Caleb Webber, John Hardy, Juan A Botía, Mina Ryten

**Affiliations:** 1 Department of Neurology, Northwestern University Feinberg School of Medicine, Chicago, IL, 60611, USA; 2 Department of Neurodegenerative Disease, Institute of Neurology, University College London, London, UK; 3 Reta Lila Weston Research Laboratories, Institute of Neurology, University College London, London, UK; 4 Istituto di Ricerca Genetica e Biomedica, Cittadella Universitaria di Cagliari, 09042, Monserrato, Sardinia, Italy; 5 Department of Biochemistry and Molecular Biology-A, Faculty of Biology, Regional Campus of International Excellence ‘Campus Mare Nostrum’, University of Murcia, Campus Espinardo, E-30100, Murcia, Spain; 6 Murcia Biomedical Research Institute (IMIB-Arrixaca), 30120, Murcia, Spain; 7 UK Dementia Research Institute at Cardiff University, Hadyn Ellis Building, Cardiff, CF24 4HQ, UK; 8 Department of Medical and Molecular Genetics, King's College London, Guy's Hospital, London, UK; 9 Department of Clinical and Movement Neurosciences, Institute of Neurology, University College London, London, UK; 10 UK Dementia Research Institute at University College London, London, UK; 11 Institute for Advanced Study, The Hong Kong University of Science and Technology, Hong Kong SAR, China; 12 Department of Information and Communications Engineering, University of Murcia, Spain; 13 NIHR Great Ormond Street Hospital Biomedical Research Centre, University College London, London, UK; 14 Genetics and Genomic Medicine, Great Ormond Street Institute of Child Health, University College London, London WC1E 6BT, UK

**Keywords:** dystonia, network analysis, medium-spiny neurons, transcriptomic analysis, synaptic transmission

## Abstract

Dystonia is a neurological disorder characterized by sustained or intermittent muscle contractions causing abnormal movements and postures, often occurring in absence of any structural brain abnormality. Psychiatric comorbidities, including anxiety, depression, obsessive-compulsive disorder and schizophrenia, are frequent in patients with dystonia. While mutations in a fast-growing number of genes have been linked to Mendelian forms of dystonia, the cellular, anatomical, and molecular basis remains unknown for most genetic forms of dystonia, as does its genetic and biological relationship to neuropsychiatric disorders. Here we applied an unbiased systems-biology approach to explore the cellular specificity of all currently known dystonia-associated genes, predict their functional relationships, and test whether dystonia and neuropsychiatric disorders share a genetic relationship. To determine the cellular specificity of dystonia-associated genes in the brain, single-nuclear transcriptomic data derived from mouse brain was used together with expression-weighted cell-type enrichment. To identify functional relationships among dystonia-associated genes, we determined the enrichment of these genes in co-expression networks constructed from 10 human brain regions. Stratified linkage-disequilibrium score regression was used to test whether co-expression modules enriched for dystonia-associated genes significantly contribute to the heritability of anxiety, major depressive disorder, obsessive-compulsive disorder, schizophrenia, and Parkinson’s disease. Dystonia-associated genes were significantly enriched in adult nigral dopaminergic neurons and striatal medium spiny neurons. Furthermore, 4 of 220 gene co-expression modules tested were significantly enriched for the dystonia-associated genes. The identified modules were derived from the substantia nigra, putamen, frontal cortex, and white matter, and were all significantly enriched for genes associated with synaptic function. Finally, we demonstrate significant enrichments of the heritability of major depressive disorder, obsessive-compulsive disorder and schizophrenia within the putamen, frontal cortex and white matter modules, and nominal enrichment of the heritability of Parkinson’s disease within the substantia nigra module. In conclusion, multiple dystonia-associated genes interact and contribute to pathogenesis likely through dysregulation of synaptic signalling in striatal medium spiny neurons, adult nigral dopaminergic neurons and frontal cortical neurons. Furthermore, the enrichment of the heritability of psychiatric disorders in the co-expression modules enriched for dystonia-associated genes indicates that psychiatric symptoms associated with dystonia are likely to be intrinsic to its pathophysiology.

## Teaser

See Busch and Klein (doi:10.1093/brain/awaa253) for a scientific commentary on this article.

Mencacci *et al*. show that multiple dystonia genes are functionally related and likely contribute to modulation of synaptic signalling in striatal, dopaminergic and frontal cortical neurons. They also demonstrate a genetic relationship between dystonia and psychiatric disorders including depression, OCD and schizophrenia.

**See Klein (doi:10.1093/brain/awaa253) for a scientific commentary on this article.**

## Introduction

The term dystonia defines a heterogeneous family of hyperkinetic movement disorders unified by their clinical manifestations, which include sustained or intermittent muscle contractions causing abnormal, often repetitive, movements, postures, or both (Albanese *et al.*, 2013). While dystonia can occur as the consequence of both focal and degenerative brain lesions, most commonly affecting the basal ganglia (Bhatia and Marsden, 1994) or cerebellum (Batla *et al.*, 2015), most patients with dystonia have normal neuroimaging findings and pathological studies have consistently shown the absence of even subtle structural abnormalities (Sharma, 2019). Thus, like epilepsy, dystonia has a dual nature, presenting both as the symptom of specific brain lesions and as a discrete disease entity.

The aetiology of dystonia occurring in the absence of structural abnormalities is unknown in most cases. Pathogenic mutations in a fast-growing number of genes have been linked to Mendelian forms of ‘idiopathic dystonia’ and appear to be an important cause of dystonia, especially in cases with paediatric onset of symptoms and/or strong family history (Balint *et al.*, 2018). Clinically, mutations in these dystonia-associated genes (hereafter termed DYT genes) produce a range of phenotypes, including isolated (dystonia occurring alone), combined (dystonia associated with myoclonus or parkinsonism) and paroxysmal forms of the condition (intermittent attacks of dystonia with normal interictal neurological examination) (Marras *et al.*, 2016; Lohmann and Klein, 2017; Zhang *et al.*, 2019), with a subset of genes capable of producing multiple clinical presentations (Friedman *et al.*, 2016; Carecchio *et al.*, 2017; Zech *et al.*, 2017; Balint *et al.*, 2019).

Genetic discoveries played a key role in conclusively pushing dystonia into the realm of neurological disorders after decades of controversy during which dystonia was considered by many to be a psychiatric condition (Lesser and Fahn, 1978). Still, dystonia not only affects motor function, but presents with additional psychiatric symptoms in ∼50–90% of patients with dystonia, including both patients with sporadic and monogenic forms (Stamelou *et al.*, 2012; Peall *et al.*, 2013, 2015; Zurowski *et al.*, 2013; Conte *et al.*, 2016). These symptoms range from depression and anxiety (Lencer *et al.*, 2009; Fabbrini *et al.*, 2010; Steinlechner *et al.*, 2017) to obsessive compulsive disorder (Voon *et al.*, 2010; Barahona-Correa *et al.*, 2011) and psychosis (Brashear *et al.*, 2012; Vijiaratnam *et al.*, 2018; Timmers *et al.*, 2019), with depression and anxiety disorders being the most common (Smit *et al.*, 2016; Berman *et al.*, 2017). Psychiatric co-morbidities often precede the onset of motor symptoms (Moraru *et al.*, 2002; Lencer *et al.*, 2009) and have a profound impact on dystonia patients’ quality of life (Smit *et al.*, 2016; Eggink *et al.*, 2019).

These observations raise the question of whether psychiatric symptoms are intrinsic to the neurobiology of dystonia, a question that is hard to address as the physiological and pathogenic roles of DYT genes are known only for a minority of genes. For instance, it is well established that the genes associated with forms of dystonia responsive to dopamine replacement (i.e. DOPA-responsive dystonias) are all involved in the synthesis and metabolism of dopamine in nigral dopaminergic neurons (Ng *et al.*, 2015; Ribot *et al.*, 2019). However, little is known about the biological function of many of the other DYT genes and how they contribute to disease pathogenesis.

The recent progress in our understanding of the genetic architecture of both dystonia and neuropsychiatric diseases, together with the increased availability of brain-related functional genomic annotations, offers a unique opportunity to robustly examine the relationship between these conditions. More specifically, whole-exome sequencing efforts have resulted in the identification of several novel monogenic causes of dystonia (described above), and genome-wide association studies (GWAS) for schizophrenia (Pardinas *et al.*, 2018), obsessive-compulsive disorder [International Obsessive Compulsive Disorder Foundation Genetics Collaborative (IOCDF-GC) and OCD Collaborative Genetics Association Studies (OCGAS), 2018], anxiety (Otowa *et al.*, 2016), and major depressive disorder (Wray *et al.*, 2018) have provided an increasingly long list of risk loci.

Thus, in this study we applied a comprehensive systems biology approach to explore the following unanswered questions: (i) which brain cells are most relevant to the pathogenesis of monogenic dystonias? (ii) do DYT genes interact and coalesce in shared cellular and molecular pathways, and if yes, in which brain regions and/or cells does this happen? and (iii) is there a genetic relationship between dystonia and neuropsychiatric disorders suggesting a shared neurobiological basis? Setting aside the DYT genes contributing to forms of DOPA-responsive dystonia, our analyses suggest that multiple dystonia-genes interact and play a fundamental role in the pathogenesis of dystonia likely through dysregulation of synaptic signalling in striatal medium spiny neurons (MSNs) and frontal cortex pyramidal neurons. Furthermore, we show that DYT genes enrich within co-expression modules that also enrich for the heritability of neuropsychiatric disorders, suggesting that the psychiatric symptoms associated with dystonia are intrinsic to its pathophysiology.

## Materials and methods

### Definition of idiopathic dystonia-related genes

Many heterogeneous monogenic disorders can present with dystonia, often amongst other neurological abnormalities, as a symptom of structural or degenerative lesions of the basal ganglia. From a biological point of view, this list includes very different conditions, including several metabolic disorders (i.e. organic acid, mitochondrial, and lysosomal storage disorders), brain metal accumulation disorders, and several neurodegenerative conditions (Fung *et al.*, 2013). Herein, we focused our analysis on monogenic dystonias that occur in the absence of any brain structural abnormality (i.e. idiopathic dystonia), hypothesizing that this group may share similar biological mechanisms, different from those of dystonias secondary to brain structural changes.

The Movement Disorders Society (MDS) has recently provided a list of the established genes clinically associated with prominent dystonia (Marras *et al.*, 2016). From this list, we included only those genes that when mutated cause prominent dystonia in the absence of imaging or neuropathological evidence of structural or degenerative abnormalities (as reported by at least two independent groups). Therefore, all DYT genes systematically associated with a radiological or pathological phenotype of basal ganglia injury, metal accumulation (i.e. iron, manganese, copper and calcium) or overt neurodegeneration (e.g. *TAF1* and other genes associated with degenerative complex dystonia-parkinsonism) were excluded. Furthermore, we included all DYT genes regardless of their most commonly associated clinical phenotype (i.e. isolated, combined and paroxysmal dystonia). To ensure our list of DYT genes was up to date, we complemented the MDS list with the additional confirmed isolated and combined dystonia genes (*ANO3* and *KMT2B*) included in the most recently published review on dystonia (Balint *et al.*, 2018). Furthermore, we included four additional genes for which an established pathogenic role in dystonia has only been recently demonstrated, namely *KCTD17* (Mencacci *et al.*, 2015; Graziola *et al.*, 2019; Marce-Grau *et al.*, 2019), *HPCA* (Charlesworth *et al.*, 2015; Atasu *et al.*, 2018), *SLC18A2* (Rilstone *et al.*, 2013; Rath *et al.*, 2017), and *DNAJC12* (Anikster *et al.*, 2017; Veenma *et al.*, 2018). As for paroxysmal dystonias, we included the confirmed genes listed in the most recent review on the topic (Zhang *et al.*, 2019). The confirmed DYT genes included in the analysis are listed in Table 1. Unconfirmed DYT genes (i.e. *CIZ1*, *COL6A3*, *RELN*) were not included in the analysis.

**Table 1:** List of dystonia-associated genes included in the analysis

Dystonia classification	Main dystonic phenotype	Gene	Inheritance	Locus	Other common clinical presentations/ additional clinical features
Combined dystonia	Dopa-responsive dystonia-parkinsonism	*DDC*	AR	None	DD, oculogyric crisis, truncal hypotonia, dysautonomia, ptosis
		*DNAJC12*	AR	None	DD, intellectual disability, HPA
		*GCH1*	AD and AR	DYT5	DD, hypotonia, spasticity and HPA in in bi-allelic mutation carriers
		*PTS*	AR	None	DD, oculogyric crisis, truncal hypotonia, dysautonomia, seizures, HPA
		*QDPR*	AR	None	DD, oculogyric crisis, truncal hypotonia, dysautonomia, seizures, HPA
		*SLC18A2*	AR	None	DD, truncal hypotonia, dysautonomia
		*SLC6A3*	AR	None	DD, oculogyric crisis, truncal hypotonia, bulbar dysfunction
		*SPR*	AR	None	DD, oculogyric crisis, truncal hypotonia, dysautonomia
		*TH*	AR	DYT5b	DD, oculogyric crisis, truncal hypotonia, dysautonomia, ptosis
	Dystonia-chorea-myoclonus	*ADCY5*	AD and AR	None	Episodic hyperkinesias triggered by sleep, truncal hypotonia
	Generalized dystonia with prominent cranial involvement	*KMT2B*	AD	DYT28	Intellectual disability, short stature
	Myoclonus-dystonia	*KCTD17*	AD	DYT26	Mild motor DD
		*SGCE*	AD	DYT11	Psychiatric co-morbidities (anxiety, obsessive compulsive disorder, alcohol addiction)
	Generalized dystonia with prominent laryngeal dystonia	*TUBB4A*	AD	DYT4	Hypomyelination with atrophy of basal ganglia and cerebellum (H-ABC)
	Generalized dystonia-parkinsonism	*PRKRA*	AR	DYT16	None
	Rapid-onset dystonia parkinsonism	*ATP1A3*	AD	DYT12	Alternating hemiplegia of childhood, Cerebellar ataxia, areflexia, pes cavus, optic atrophy, and sensorineural hearing loss (CAPOS) syndrome, paroxysmal limb dystonia
Isolated dystonia	Adult-onset cranio-cervical dystonia	*ANO3*	AD	DYT24	Subcortical myoclonus, prominent dystonic tremor
		*GNAL*	AD and AR	DYT25	None
	Early-onset generalized dystonia	*HPCA*	AR	DYT2	Learning disabilities, DD
		*THAP1*	AD and AR	DYT6	None
		*TOR1A*	AD	DYT1	Severe arthrogryposis in bi-allelic mutation carriers
Paroxysmal dystonia	Paroxysmal exercise-induced dystonia	*SLC2A1*	AD	DYT9 or DYT18	GLUT1-deficiency syndrome (DD, spasticity, microcephaly, ataxia, epilepsy)
	Paroxysmal kinesigenic dystonia	*KCNA1*	AD	n/a	Paroxysmal ataxia with interictal myokymia
		*PRRT2*	AD	DYT10 or DTY19	Benign familial infantile epilepsy, hemiplegic migraine, paroxysmal torticollis of infancy
		*SCN8A*	AD	None	Benign familial infantile seizures, early infantile epileptic encephalopathy
	Paroxysmal non-kinesigenic dystonia	*KCNMA1*	AD	None	Epilepsy, DD, cerebellar ataxia
		*PNKD*	AD	DYT8	None
	Paroxysmal torticollis of infancy	*CACNA1A*	AD	None	Paroxysmal ataxia with interictal dystonia, hemiplegic migraine, SCA6, early infantile epileptic encephalopathy

Genes have been grouped by their overall dystonia classification and main dystonic phenotype. Within these groupings, genes are sorted alphabetically. AD = autosomal dominant; AR = autosomal recessive; DD = developmental delay; HPA = hyperphenylalanaemia; SCA = spinocerebellar ataxia.

### Expression-weighted cell-type enrichment

Expression-weighted cell-type enrichment (EWCE) (see ‘URLs’ section) was used to determine whether DYT genes have higher expression within particular brain-related cell types than would be expected by chance (Skene and Grant, 2016). As our input we used (i) the list of DYT genes as defined above; and (ii) specificity values calculated for level 1 cell types from two independent single-cell/nuclei RNA-sequencing datasets. These datasets included (i) mouse single-cell RNA sequencing from the Karolinska superset, which includes cell types from the neocortex, hippocampus, hypothalamus, striatum and midbrain (see ‘URLs’ section) (Skene *et al.*, 2018); and (ii) human single-nuclei RNA sequencing from the substantia nigra (Agarwal *et al.*, submitted for publication, accessed via GEO; GEO accession: GSE140231; see ‘URLs’ section). Specificity values (i.e. proportion of total expression of a gene in one cell type compared to all others) for the mouse Karolinska superset had been previously published (Skene *et al.*, 2018). For the GEO-derived dataset, however, the cell-type specificity of each gene was estimated using substantia nigra-derived read count values together with the ‘generate.celltype.data()’ function of the EWCE package. The generated specificity matrix is available online via the MarkerGenes package (see ‘URLs’ section). EWCE with the target list was run with 100 000 bootstrap replicates, which were sampled from a background list of expressed genes (when using mouse-derived data, this background list excluded all genes without a 1:1 mouse:human orthologue). We additionally controlled for transcript length and GC content biases by selecting bootstrap lists with comparable properties to the target list. Data are displayed as standard deviations from the mean, and any values <0, which reflect a depletion of expression, are displayed as 0. *P*-values were corrected for multiple testing using the Benjamini-Hochberg method over all cell types in each dataset.

### Weighted gene co-expression analysis

We generated gene co-expression networks (GCNs) for CNS tissue-specific transcriptomic data generated by the UK Brain Expression Consortium (UKBEC) (Ramasamy *et al.*, 2014) and Genotype-Tissue Expression Consortium (GTEx) (GTEx Consortium, 2015) (Version 6; www.gtexportal.org). In total, 57 gene-level expression datasets across an equal number of tissues were used with the weighted gene co-expression network analysis (WGCNA) R package with k-means adjustment to generate tissue-specific networks (Langfelder and Horvath, 2008; Langfelder *et al.*, 2008; Botia *et al.*, 2017). For each tissue, a ‘signed’ GCN was constructed by creating a signed Topological Overlap Measure (TOM) matrix based on Pearson correlation. Gene modules were created by hierarchical clustering based on a 1 − TOM dissimilarity matrix. The results of the initial hierarchical clustering were post-processed using the k-means clustering search method with 30 iterations. All co-expression networks are available online via the CoExp website (www.rytenlab.com/coexp/Run/Catalog/) or CoExpNets package (https://github.com/juanbot/CoExpNets) to enable use with third-party software.

To assess module preservation within and between datasets we performed a preservation analysis based on WGCNA using the *Z* summary statistic to evaluate preservation (Langfelder *et al.*, 2011). As per Langfelder *et al.*, if the *Z* summary statistic is >10, there is strong evidence of module preservation; if 2 < *Z* summary statistic <10, there is weak to moderate evidence of preservations; and finally, if the *Z* summary statistic is <2, there is no evidence of preservation.

Gene modules were functionally annotated with gProfileR R package using Gene Ontology (GO) database without Electronic Inferred Annotations (EIA) and accounting for multiple testing with gSCS (Reimand *et al.*, 2007). Additional functional annotations for modules of interest were generated using the web server SynGO (https://www.syngoportal.org/), which provides an expert-curated resource for synapse function and gene enrichment analysis (Koopmans *et al.*, 2019). All gene set enrichment analyses were performed using default settings, with no annotation filters applied and a minimum gene count of three for ontology terms to be included in the overrepresentation analysis.

### Stratified linkage disequilibrium score regression

Stratified linkage disequilibrium score regression (LDSC) (see ‘URLs’ section) (Finucane *et al.*, 2015) was used to test whether co-expression modules enriched for DYT genes significantly contributed to the common single nucleotide polymorphism (SNP) heritability of four neuropsychiatric disorders (anxiety, major depressive disorder, obsessive compulsive disorder, and schizophrenia) and one neurodegenerative disorder (Parkinson’s disease) (Table 2). Co-expression modules from UKBEC and GTEx were downloaded using the CoExpNets package (see ‘URLs’ section). As previously performed, modules were filtered to include only genes with module membership ≥0.5 (Reynolds *et al.*, 2019). Furthermore, gene coordinates were extended by 100 kb upstream and downstream of their transcription start and end site, in order to capture regulatory elements that might contribute to disease heritability (Finucane *et al.*, 2018).

**Table 2:** Summary of GWAS datasets

Disease	Cases, *n*	Controls, *n*	Reference
Anxiety	7016	14 745	Otowa *et al.* (2016)
Major depressive disorder (excluding 23andMe contributions)	59 851	113 154	Wray *et al.* (2018)
Obsessive compulsive disorder	2688	7037	International Obsessive Compulsive Disorder Foundation Genetics Collaborative (IOCDF-GC) and OCD Collaborative Genetics Association Studies (OCGAS) (2018)
Parkinson’s disease (excluding 23andMe contributions)	33 674 (18 618 proxy cases from UK Biobank)	449 037	Nalls *et al.* (2019)
Schizophrenia	40 675	64 643	Pardinas *et al.* (2018)

All annotations were constructed in a binary format (1 if the SNP was present within the annotation and 0 if not), using all SNPs with a minor allele frequency >5%. Annotations were then added individually to the baseline model of 53 annotations provided by Finucane *et al*. (2015). (version 1.2, see ‘URLs’ section), comprising genome-wide annotations reflecting genetic architecture. HapMap Project Phase 3 (HapMap3) (Altshuler *et al.*, 2010) SNPs and 1000 Genomes Project (Abecasis *et al.*, 2012) Phase 3 European population SNPs were used for the regression and LD reference panels, respectively. The MHC region was excluded from all analyses because of the complex and long-range linkage disequilibrium patterns in this region. For all stratified LDSC analyses, we report a one-tailed *P*-value (coefficient *P*-value) based on the coefficient *z*-score outputted by stratified LDSC. A one-tailed test was used as we were only interested in annotation categories with a significantly positive contribution to trait heritability, conditional upon the baseline model. Multiple test correction was performed within a network. Thus, to account for multiple tests, the Bonferroni significance threshold was set to 0.05 divided by the number of modules for each co-expression network (four for UKBEC; three for GTEx) and the number of GWASs run. Nominal enrichments were defined as those enrichments that did not pass the Bonferroni cut-off, but passed *P < *0.05.

### URLs

CoExp website, www.rytenlab.com/coexp/Run/Catalog/; CoExpNets package, https://github.com/juanbot/CoExpNets; EWCE, https://github.com/NathanSkene/EWCE; Human substantia nigra single-nuclei RNA sequencing, https://www.ncbi.nlm.nih.gov/geo/query/acc.cgi?acc=GSE140231; Karolinska single-cell RNA sequencing superset, https://github.com/NathanSkene/MAGMA_Celltyping; LDSC, https://github.com/bulik/ldsc; baseline LDSC annotations, https://data.broadinstitute.org/alkesgroup/LDSCORE/; MarkerGenes, https://github.com/RHReynolds/MarkerGenes.

### Data availability

**Figure 1:**
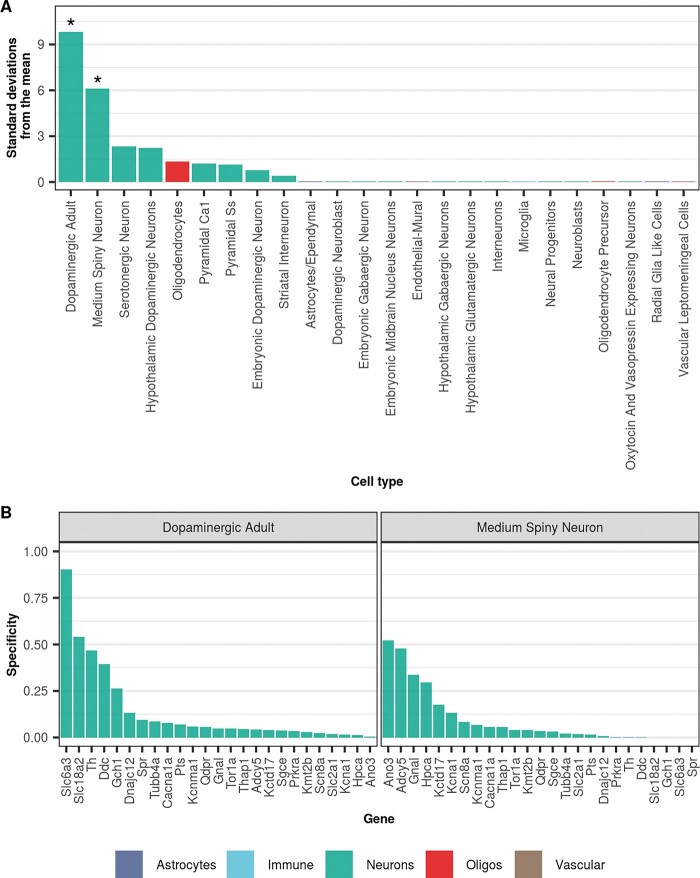
**Dystonia-associated genes are highly expressed in dopaminergic neurons and MSNs from mouse.** (**A**) Enrichment of dystonia-associated genes in level 1 cell types from the Karolinska superset was determined using EWCE. Standard deviations from the mean indicate the distance of the mean expression of the target list from the mean expression of the bootstrap replicates. Asterisks denote significance at *P < *0.05 after correcting for multiple testing with the Benjamini-Hochberg method over all level 1 cell types. Numerical results are reported in Supplementary Table 1. (**B**) Plot of specificity values for all dystonia-associated genes within adult nigral dopaminergic neurons and MSNs (level 1 cell types from the Karolinska single-cell RNA-sequencing superset). Specificity values were derived from Skene *et al.* (2018) who calculated specificity by dividing the mean expression of a gene in one cell type by the mean expression in all cell types. In other words, specificity is the proportion of a gene’s total expression attributable to one cell type, with a value of 0 meaning a gene is not expressed in that cell type and a value of 1 meaning that a gene is only expressed in that cell type. In both plots, cell types are coloured by the overall class they belong to (e.g. astrocyte, neuron, oligodendrocyte, etc.).

**Figure 2:**
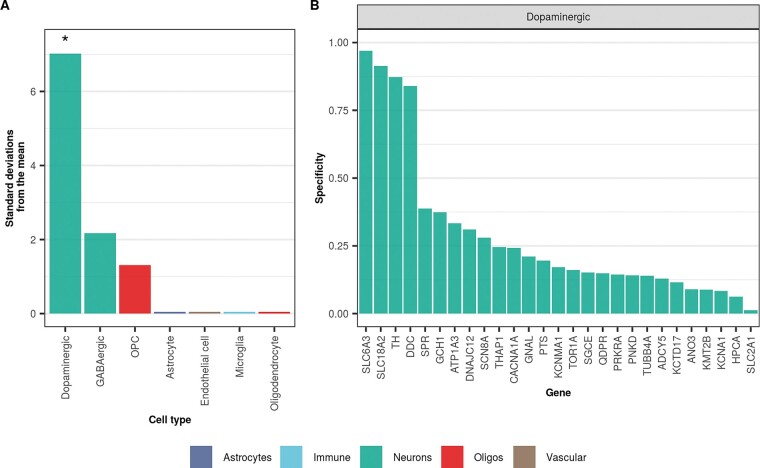
**Dystonia-associated genes are highly expressed in human dopaminergic neurons.** (**A**) Enrichment of dystonia-associated genes in level 1 cell types from human-derived substantia nigra single-nuclei RNA-sequencing data was determined using EWCE. Standard deviations from the mean indicate the distance of the mean expression of the target list from the mean expression of the bootstrap replicates. Asterisks denote significance at *P < *0.05 after correcting for multiple testing with the Benjamini-Hochberg method over all level 1 cell types. Numerical results are reported in Supplementary Table 2. (**B**) Plot of specificity values for all dystonia-associated genes within dopaminergic neurons (level 1 cell type from human-derived substantia nigra single-nuclei RNA-sequencing data). Specificity values represent the proportion of a gene’s total expression attributable to one cell type, with a value of 0 meaning a gene is not expressed in that cell type and a value of 1 meaning that a gene is only expressed in that cell type. In both plots, cell types are coloured by the overall class they belong to (e.g. astrocyte, neuron, oligodendrocyte, etc.).

All co-expression networks are openly available online via the CoExp website (www.rytenlab.com/coexp/Run/Catalog/) or CoExpNets package (https://github.com/juanbot/CoExpNets). Raw data used to generate the specificity matrix derived from human substantia nigra single-nuclei sequencing is openly available online at https://www.ncbi.nlm.nih.gov/geo/query/acc.cgi? acc=GSE140231; the generated specificity matrix is openly available at https://github.com/RHReynolds/MarkerGenes. The specificity matrix derived from the Karolinska superset is openly available at https://github.com/NathanSkene/MAGMA_Celltyping. Data used to generate Figs 1, 2, 4–6 and Supplementary Figs 1–3 are available within the Supplementary material.

**Figure 3:**
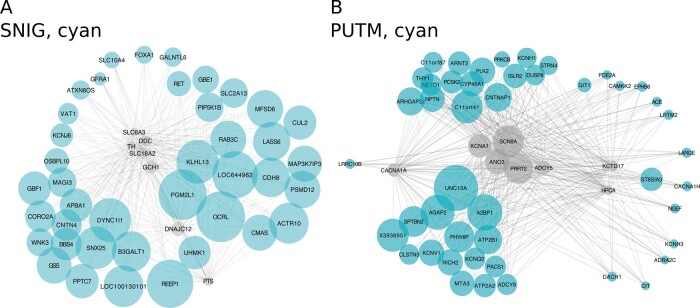
**Gene co-expression modules enriched for dystonia genes in the substantia nigra and putamen.** The substantia nigra (SNIG) ‘cyan’ (**A**) and putamen (PUTM) ‘cyan’ (**B**) dystonia-linked UKBEC modules visualized using ‘bottom-up’ plots. In each case, the dystonia genes within the modules are depicted as grey nodes with their most connected genes, as determined by the Topology Overlap Measure, depicted in cyan (to a maximum of seven genes per seed). Node size reflects connectivity. Plots were generated using Cytoscape 3.5.1 and the Edge-weighted Spring Embedded layout algorithm was used for rendering to a 2D canvas.

## Results

### Dystonia genes are highly and specifically expressed in midbrain dopaminergic and striatal medium spiny neurons

In the first instance, we wanted to study the cellular specificity of DYT genes across a broad range of brain regions, with the aim of identifying the cell types most likely involved in primary pathology. Using EWCE analysis and single-cell gene expression profiling of the mouse brain (Karolinska Institute brain superset from the Linnarsson group), which included cell types from the neocortex, hippocampus, hypothalamus, striatum and midbrain, we demonstrated that DYT genes are significantly enriched in two cell types, namely adult nigral dopaminergic neurons [false discovery rate (FDR)-adjusted *P*-value < 0.00001] and striatal MSNs (FDR-adjusted *P = *0.00156; Fig. 1A and Supplementary Table 1).

The enrichment within adult nigral dopaminergic neurons was largely driven by genes responsible for forms of dystonia responsive to dopaminergic therapies (i.e. DOPA-responsive dystonias) (Ng *et al.*, 2015). In fact, we noted that *SLC6A3*, *SLC18A2*, *TH*, *DDC*, and *GCH1*, had the highest specificity values in adult nigral dopaminergic neurons (Fig. 1B and Supplementary Table 1).

*ANO3*, *ADCY5*, *GNAL*, and *KCTD17* were the genes with the highest specificity values in striatal MSNs. Amongst these, *ADCY5* and *GNAL* had specificity values more than 4-fold higher in MSNs than in other cell types, indicating almost exclusive expression in this cell type (Supplementary Fig. 1 and Supplementary Table 1). While some of the genes driving this enrichment have an established function in MSNs (i.e. *GNAL* and *ADCY5* are involved in striatal dopaminergic and adenosinergic post-receptor signalling) (Goodchild *et al.*, 2013), *ANO3* and *KCTD17* have not been studied in this context and their physiological role in MSNs is currently unknown.

To ensure our results were robust to species differences, we performed EWCE using single-nuclei gene expression profiling from the human substantia nigra (Agarwal *et al.*, submitted for publication, accessed via GEO; GEO accession: GSE140231; see ‘URLs’ section). We found that DYT genes are significantly enriched in human dopaminergic neurons (FDR-adjusted *P*-value < 0.00001), confirming the enrichment observed using mouse-derived single-cell data (Fig. 2A and Supplementary Table 2). As in mouse dopaminergic neurons, *SLC6A3*, *SLC18A2*, *TH*, *DDC*, and *GCH1* had the highest specificity values in human dopaminergic neurons (Fig. 2B and Supplementary Table 2) and, in general, there was a strong positive correlation between DYT gene specificity values in dopaminergic neurons derived from mouse and human data (spearman’s rho = 0.85; *P*-value < 0.001; Supplementary Fig. 2).

### Gene co-expression network analysis identifies dystonia-enriched modules

Commonalities in the cellular specificity of DYT genes suggested the possibility that a subset of DYT genes may be functionally related. However, EWCE analysis was based on cell-specific gene expression data derived from mouse and did not consider correlations in gene expression. Co-expression networks have proven to be an efficient means of identifying hidden functional relationships between genes of interest (Oldham *et al.*, 2008; Kelley *et al.*, 2018). Thus, to explore the possibility that a subset of DYT genes are functionally related, we tested for the enrichment of DYT genes within co-expression networks generated from 10 regions of the human brain using transcriptomic data from UKBEC (Ramasamy *et al.*, 2014). Brain regions included: cerebellar cortex, frontal cortex, hippocampus, inferior olivary nucleus, occipital cortex, putamen (dissected at the level of the anterior commissure), substantia nigra, temporal cortex, thalamus and intralobular white matter.

Amongst the 220 gene co-expression modules tested, we identified four modules in which DYT genes were significantly enriched (FDR-adjusted *P*-value < 0.05; Supplementary Table 3). These four modules derived from the substantia nigra (‘cyan’ module), putamen (‘cyan’ module), frontal cortex (‘lightyellow’ module) and white matter (‘blue’ module) networks. No enrichment of DYT genes was observed in any of the other brain regions. To replicate these findings, we tested for enrichment of DYT genes in co-expression networks generated using transcriptomic data from the GTEx Project; only co-expression networks derived from frontal cortex, putamen and substantia nigra were used. Amongst the co-expression modules tested, DYT genes enriched in three modules: frontal cortex ‘turquoise’ module, putamen ‘blue’ module and substantia nigra ‘darkorange2’ module.

The overlap of DYT genes with the substantia nigra ‘cyan’ module was driven by seven DYT genes (*GCH1*, *TH*, *SLC6A3*, *DNAJC12*, *DDC*, *SLC18A2*, *PTS*; FDR-adjusted *P = *0.0409) (Fig. 3A). This finding was replicated using GTEx-derived co-expression networks, with a significant enrichment of DYT genes in the substantia nigra ‘darkorange2’ module (FDR-adjusted *P = *0.005) driven by an overlapping set of six genes, namely *GCH1*, *TH*, *SLC6A3*, *DDC*, *SLC18A2* and *PTS*. Importantly, mutations in all DYT genes enriched in these modules cause DOPA-responsive dystonias and are well known to be functionally related to dopamine synthesis and/or metabolism.

The most significant enrichment of DYT genes was detected in a putamen gene co-expression module. Of the 28 DYT genes tested, eight overlapped with the ‘cyan’ module in this tissue (*ADCY5*, *ANO3*, *KCTD17*, *HPCA*, *PRRT2*, *SCN8A*, *KCNA1*, *CACNA1A;* FDR-adjusted *P = *0.0001) (Fig. 3B). This module included established cellular markers of MSNs, including *DRD1*, *DRD2*, *ADORA2A* and *PPP1R1B*, indicating the module captures the expression signature of these neurons. Again, this finding was supported by replication in GTEx-derived co-expression networks (putamen ‘blue’ module; FDR-adjusted *P = *0.026), with the enrichment driven by an overlapping set of six DYT genes (*ADCY5*, *KCTD17*, *HPCA*, *PRRT2*, *PNKD*, and *CACNA1A*).

Significant enrichment of DYT genes was also detected in the white matter ‘blue’ module (overlap of eight genes, *PRRT2*, *PNKD*, *SCN8A*, *KCNA1*, *ATP1A3*, *ANO3*, *KCTD17*, *HPCA*; FDR-adjusted *P = *0.0203) and the ‘light-yellow’ frontal cortex module (overlap of six genes, *ANO3*, *GNAL*, *KCTD17*, *HPCA*, *KCNMA1*, *CACNA1A*; FDR-adjusted *P = *0.0203). Both modules were predicted to be expression signatures of cortical pyramidal neurons. Where analysis was possible due to data availability (white matter tissue is not available in GTEx), we noted that once again the findings in frontal cortex were replicated in GTEx-derived co-expression networks (frontal cortex ‘turquoise’ module; *P = *0.0289).

Functional annotation showed that all four UKBEC dystonia-linked modules were significantly enriched for genes associated with neuronal synaptic transmission (putamen ‘cyan’ module, corrected *P = *3.44 × 10^−8^; white matter ‘blue’ module *P = *3.4 × 10^−52^; frontal cortex ‘lightyellow’ module, *P = *9.82 × 10^−7^; substantia nigra cyan module, *P = *0.0247). Furthermore, in the case of both the ‘blue’ and ‘lightyellow’ modules we found significant enrichment of terms relating to neurodevelopment, namely neuron projection development (white matter ‘blue’ module, *P = *4.96 × 10^−26^) and nervous system development (frontal cortex ‘lightyellow’ module, nervous system development, *P = *1.14 × 10^−5^). Finally, in the case of the ‘cyan’ putamen module, the most over-represented terms related to metal ion transmembrane transport, indicating an enrichment of genes coding for ion channels.

**Figure 4:**
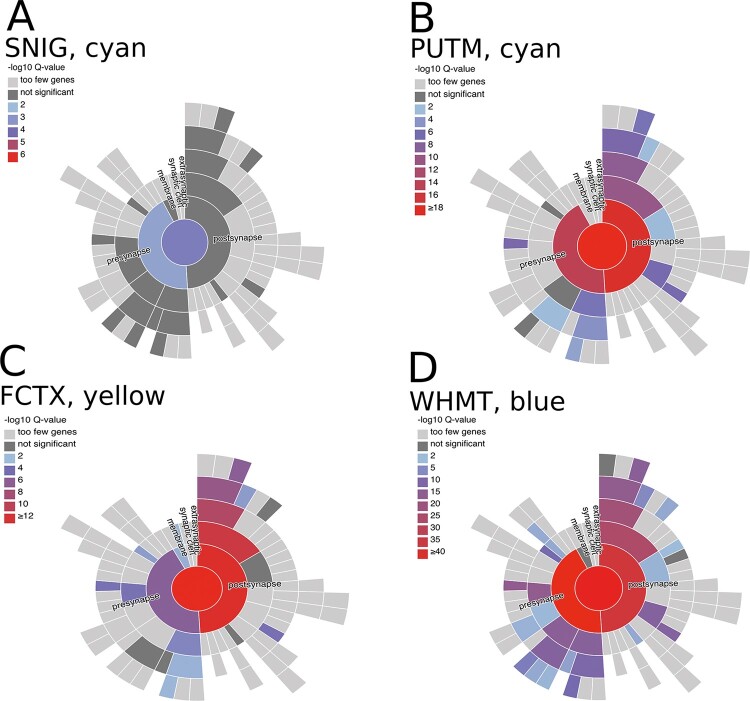
**Variable enrichment of genes associated with presynaptic and postsynaptic structures within dystonia-linked UKBEC co-expression modules.** Visualizations of the enrichment of SynGO ontology terms for synaptic location within the dystonia-linked UKBEC modules (the substantia nigra ‘cyan’, putamen ‘cyan’, frontal cortex ‘lightyellow’ and white matter ‘blue’ modules) provided by the SynGO web resource (https://www.syngoportal.org/index.html). The hierarchical structure of SynGO terms is represented by concentric rings with the most specific terms placed peripherally. The colour coding of the terms is based on the enrichment q-values. FCTX = frontal cortex; PUTM = putamen; SNIG = substantia nigra; WHMT = white matter.

To investigate the significance of these modules further, we sought to identify whether the enrichment of genes relating to synaptic transmission was driven by genes involved in postsynaptic as compared to presynaptic structures. To address this question, we checked for enrichment of genes that have been reliably associated with a specific synaptic structure using the recently released SynGO database (Koopmans *et al.*, 2019). This demonstrated significant enrichment of genes associated with both presynaptic and postsynaptic structures amongst those contained within all four modules of interest (namely the substantia nigra ‘cyan’, putamen ‘cyan’, frontal cortex ‘lightyellow’ and white matter ‘blue’ modules). However, whereas the substantia nigra ‘cyan’ and white matter ‘blue’ modules were more significantly enriched for genes associated with presynaptic structures, the putamen ‘cyan’ and frontal cortex ‘lightyellow’ modules showed more significant enrichments for genes associated with postsynaptic structures (Fig. 4 and Supplementary Table 4).

### Dystonia-linked modules in the putamen and substantia nigra represent expression signatures unique to these tissues

**Figure 5:**
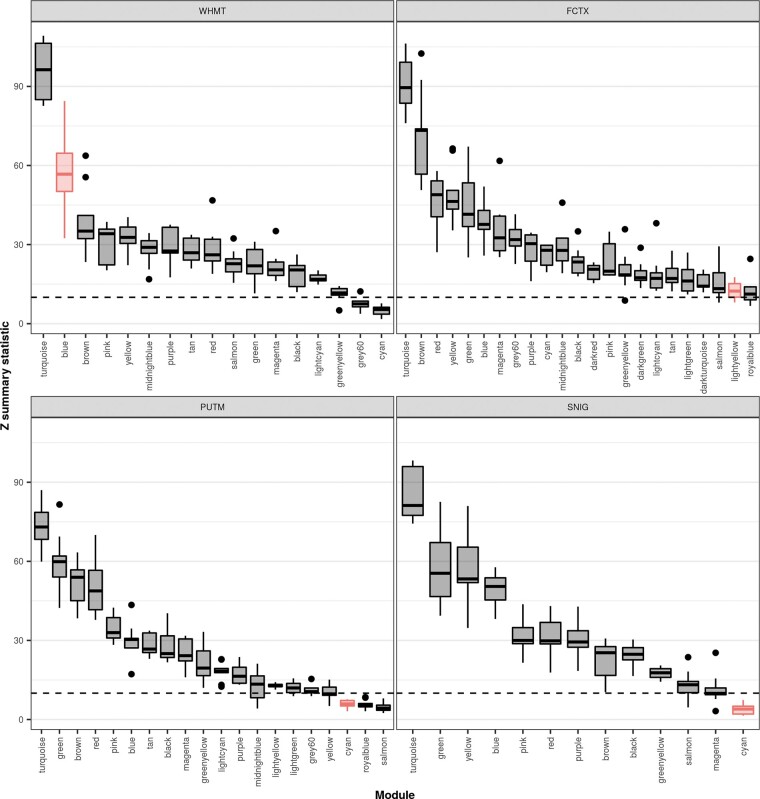
**Module preservation within UKBEC tissues containing dystonia-linked modules.** Plot of module preservation for each module within UKBEC tissues containing dystonia-linked modules. Module preservation, as denoted by the *Z* summary statistic, was determined using a WGCNA-based preservation analysis applied across all 10 UKBEC co-expression networks. Thus, each box plot represents the preservation of the named module within the labelled tissue across the remaining nine tissues. The black dashed lines indicate the threshold for strong evidence of module preservation (*Z* summary statistic > 10), as defined by Langfelder *et al.* (2011). Modules enriched with dystonia genes are highlighted in red.

We noted high overlaps amongst the DYT genes that enriched in putamen, frontal cortex and white matter co-expression modules, with three DYT genes appearing in all three of the relevant modules (*ANO3*, *HPCA* and *KCTD17*), and a further four DYT genes appearing in at least two of these modules (*KCNA1*, *CACNA1A*, *PRRT2*, and *SCN8A*). As expected, given their well-defined role in nigral dopamine synthesis, the genes clustering in the substantia nigra module were found to enrich exclusively in that tissue. We extended this analysis by calculating module preservation statistics to assess how similar the four dystonia-linked modules were to all co-expression modules identified in the UKBEC dataset. Interestingly, we found that while the white matter ‘blue’ and frontal cortex ‘lightyellow’ modules showed strong evidence of preservation in other brain regions, the substantia nigra and putamen ‘cyan’ modules showed weak evidence of preservation (white matter ‘blue’ module, median *Z* summary statistic = 56.71; frontal cortex ‘lightyellow’ module, median *Z* summary statistic = 12.35; putamen ‘cyan’ module, mean *Z* summary statistic = 5.93; substantia nigra ‘cyan’ module, median *Z* summary statistic = 3.95) (Fig. 5 and Supplementary Table 5). This indicates that the dystonia-linked putamen and substantia nigra co-expression modules represent expression signatures specific to these brain tissues. Furthermore, these results suggest that while the DYT genes contained within these modules may be widely expressed, they have gene-gene interactions that are tissue-specific in nature.

### Heritability of psychiatric disorders is significantly enriched in dystonia-linked modules

The identification of four UKBEC-derived co-expression modules enriched for DYT genes (substantia nigra ‘cyan’, putamen ‘cyan’, frontal cortex ‘lightyellow’ and white matter ‘blue’) provided an opportunity to investigate commonalities in the underlying genetic architecture of dystonia and a range of neuropsychiatric disorders noted to have a high prevalence amongst individuals with the condition. Using stratified LDSC we tested whether genes assigned to the modules with high confidence (based on a module membership of ≥ 0.5) significantly contributed to the common SNP heritability of anxiety, major depressive disorder, obsessive-compulsive disorder, and schizophrenia. Given that in recent years dystonia has been viewed primarily as a movement disorder with significant phenotypic overlap with Parkinson’s disease (Shetty *et al.*, 2019), we extended our analysis to include this condition.

**Figure 6:**
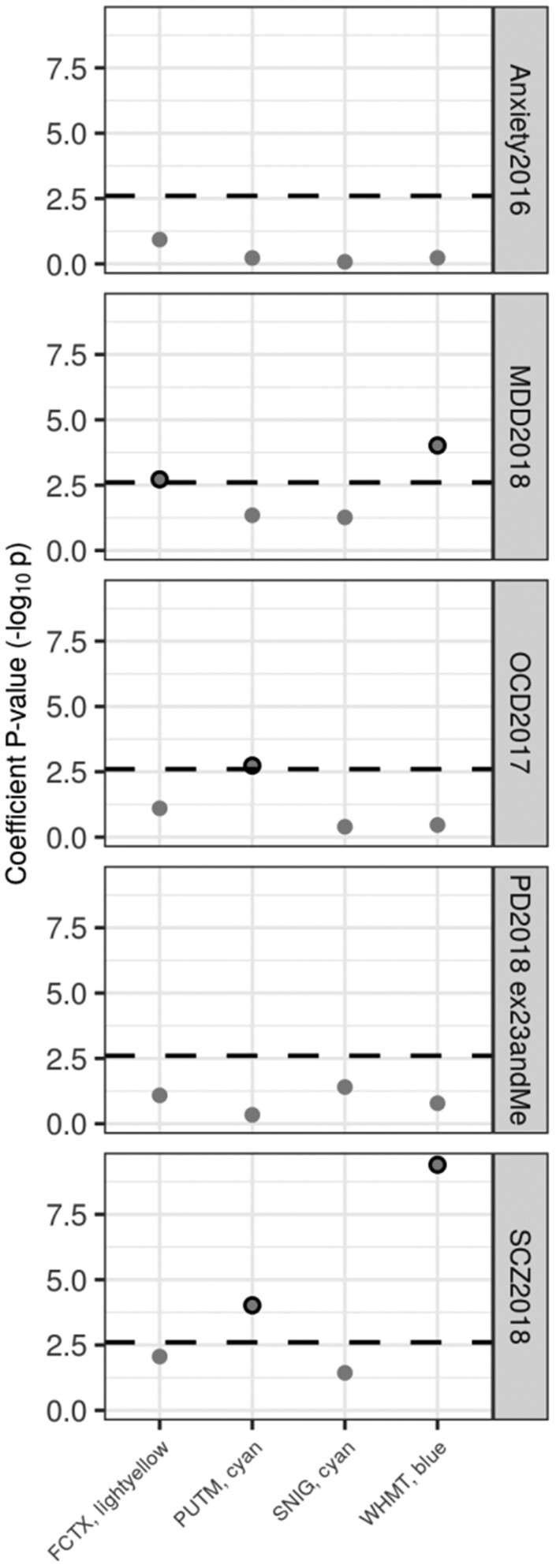
**Enrichment of disease heritability within dystonia-linked UKBEC co-expression modules.** Stratified LDSC using UKBEC co-expression modules. The black dashed lines indicate the cut-off for Bonferroni significance [*P < *0.05 / (4 × 5)]. Bonferroni-significant results are marked with black borders. Numerical results are reported in Supplementary Table 6. FCTX = frontal cortex; MDD = major depressive disorder; OCD = obsessive compulsive disorder; PD = Parkinson’s disease; PUTM = putamen; SCZ = schizophrenia; SNIG = substantia nigra; WHMT = white matter.

Stratified LDSC demonstrated a significant enrichment of obsessive-compulsive disorder and schizophrenia heritability in the putamen ‘cyan’ module (obsessive-compulsive disorder, coefficient *P = *0.0018; schizophrenia, coefficient *P*-value = 9.57 × 10^−5^); a significant enrichment of major depressive disorder and schizophrenia heritability in the white matter ‘blue’ module (major depressive disorder, coefficient *P = *9.56 × 10^−5^; schizophrenia, coefficient *P = *4.00 × 10^−10^); and a significant enrichment of major depressive disorder heritability in the frontal cortex ‘lightyellow’ module (coefficient *P* = 0.0019; Fig. 6 and Supplementary Table 6). Additionally, we observed several nominal heritability enrichments, defined as those enrichments in co-expression modules that did not pass the Bonferroni cut-off [*P < *0.05 / (4 × 5)], but passed *P < *0.05 (Supplementary Table 6). This included a nominal enrichment of Parkinson’s disease heritability in the substantia nigra ‘cyan’ module (coefficient *P* = 0.040). To assess the potential relevance of these nominal enrichments, we tested if any could be replicated in the GTEx-derived co-expression modules enriched for DYT genes. While we were unable to replicate the enrichment of Parkinson's disease heritability in the substantia nigra (GTEx substantia nigra ‘darkorange2’, coefficient *P* = 0.066), the following findings were replicated: first, an enrichment of schizophrenia heritability in the frontal cortex module (UKBEC frontal cortex ‘lightyellow’, coefficient *P* = 0.0087; GTEx frontal cortex ‘turquoise’, coefficient *P* = 3.81 × 10^−10^); and second, an enrichment of major depressive disorder heritability in the putamen module (UKBEC putamen ‘cyan’, coefficient *P* = 0.045; GTEx putamen ‘blue’, coefficient *P* = 4.68 × 10^−5^) (Supplementary Fig. 3 and Supplementary Table 6).

## Discussion

While there has been remarkable progress in our understanding of the genetic structure of dystonia, the anatomical, cellular, and molecular basis remains unknown for most genetic forms of dystonia, as does its genetic and biological relationship to neuropsychiatric disorders. Using a systems biology approach leveraging our current understanding of the genetic basis of dystonia and neuropsychiatric disease, we show that: (i) the expression of the currently known DYT genes is significantly enriched in mouse adult nigral dopaminergic neurons and striatal MSNs and in human dopaminergic neurons; (ii) multiple DYT genes are highly co-expressed with each other in multiple brain regions relevant to dystonia functional neuroanatomy, including the substantia nigra, putamen, frontal cortex and white matter; (iii) and finally, there is evidence of a genetic relationship between dystonia and complex neuropsychiatric diseases.

We found that the enrichment of DYT genes in mouse and human dopaminergic neurons detected using EWCE analysis was largely driven by genes that are involved in various steps of dopamine synthesis and metabolism in nigral dopaminergic neurons and when mutated are responsible for DOPA-responsive dystonias (Ribot *et al.*, 2019). Similarly, WGCNA-based analyses showed that the same genes clustered together in a single co-expression module specific to the substantia nigra (‘cyan’ module). These results are consistent with expectation, thus highlighting the reliability of our hypothesis-free approach in identifying disease-relevant cell types and tissue-specific gene-gene interactions amongst DYT genes. With this in mind, we note that none of the ‘non-DOPA-responsive’ DYT genes were highly specific to dopaminergic neurons, nor did we find that any co-clustered with the DOPA-responsive DYT genes in the substantia nigra ‘cyan’ co-expression module. Together, these results support the growing view that DOPA-responsive dystonias should be considered a distinct subgroup not only from a clinical perspective but also from a biological point of view.

Other neuronal cell types highlighted by our EWCE- and WGCNA-based analyses include MSNs, which constitute 95% of the cellular population of the putamen (Gerfen and Surmeier, 2011). EWCE analysis showed that four DYT genes, namely *ADCY5*, *GNAL*, *ANO3*, and *KCTD17*, had the highest specificity of expression in MSNs, suggesting they were the DYT genes driving the enrichment in MSNs. Importantly, WGCNA results went beyond simply suggesting a role for individual DYT genes within MSNs and indicated a functional interaction of multiple DYT genes in a single convergent pathway. Indeed, we observed eight DYT genes, including three of four DYT genes with the highest specificity in MSNs (*ADCY5*, *ANO3*, and *KCTD17*) and five others that were not highlighted by EWCE analysis (*CACNA1A*, *SCN8A*, *KCNA1*, *PRRT2*, *HPCA*), to be co-expressed in the putamen ‘cyan’ module (the top module for DYT gene enrichment across all tested co-expression modules). This co-expression module also contained several established MSN-specific expression markers, including: *DRD1*, *DRD2* and *ADORA2A*, which encode the striatal dopamine and adenosine receptors; and *PPP1R1B*, which encodes DARPP-32, the universal marker of MSNs and part of the signalling cascade downstream of dopaminergic and adenosinergic receptor activation (Fienberg *et al.*, 1998). In summary, these results support a functional interaction and biological convergence of multiple DYT genes in MSNs. This biological convergence is noteworthy, given that the function of several DYT genes found clustering in the putamen ‘cyan’ co-expression module (e.g. *KCTD17*, *HPCA*, *ANO3*) is poorly characterized, especially in the context of MSN biology.

To understand the biological function of DYT genes better, all dystonia-linked co-expression modules were functionally annotated using Gene Ontology terms. This functional annotation revealed that all four dystonia-linked co-expression modules were enriched for genes associated with synaptic function (in particular, genes associated with presynaptic and postsynaptic structures), suggesting that DYT genes found within these modules are also involved in synaptic function. Notably, in the putamen-specific ‘cyan’ co-expression module the enrichment for genes associated with postsynaptic structures yield a lower *P*-value than that for genes associated with presynaptic structures. Given the central role of MSNs in receiving and gating synaptic inputs from cortical and thalamic glutamatergic neurons, this suggests that disruption of postsynaptic function in MSNs through mutations in multiple DYT genes may be important in dystonia pathogenesis. In support of this hypothesis are the following observations: (i) abnormal plasticity and loss of synaptic downscaling at cortico-striatal synapses has been shown to be a dystonia endophenotype shared by different genetic animal models of dystonia (Martella *et al.*, 2014; Calabresi *et al.*, 2016; Maltese *et al.*, 2017; Zakirova *et al.*, 2018; Yu-Taeger *et al.*, 2020); and (ii) *ADCY5* and *GNAL*, two DYT genes, form part of the signalling transduction machinery in response to stimulation of dopaminergic and adenosinergic signalling in MSNs (Herve, 2011; Goodchild *et al.*, 2013; Pelosi *et al.*, 2017). Finally, evidence from genetic studies in *Drosophila* support *in vivo* our findings implicating new dystonia genes in postsynaptic signalling. Indeed, both *inc* and *Nca* (homologues of *KCTD17* and *HPCA*, respectively) were recently found to modulate sleep in *Drosophila*, specifically through disruption of dopaminergic postsynaptic pathways (Pfeiffenberger and Allada, 2012; Chen *et al.*, 2019; Kikuma *et al.*, 2019).

These findings have significant clinical implications, particularly for classification of dystonias. Currently, genetic forms of dystonia are primarily classified based on their clinical presentation. However, there is significant variability and pleiotropy in the clinical presentation of DYT mutation carriers (Table 1), which makes the exact assignment of a DYT mutation to a specific class of dystonia a hard task. Additionally, it is debatable whether grouping patients based on their clinical presentation correctly mirrors the underlying neuroanatomical or biological substrates of different types of dystonia. Importantly, the phenotypes associated with mutations in the DYT genes enriched in the putamen ‘cyan’ module belonged to different clinical subtypes of dystonia, namely isolated dystonia (*ANO3* and *HPCA*), combined dystonia (*KCTD17* and *ADCY5*), and paroxysmal dystonia (*KCNA1*, *CACNA1A*, *PRRT2* and *SCN8A*). Furthermore, the putamen ‘cyan’ module contained several other genes linked to monogenic hyperkinetic movement disorders, including genes associated with inherited forms of chorea (*PDE2A*, *GPR88*, *HTT*, *VPS13A*, *JPH3*) and dyskinetic epileptic encephalopathies (*GNB1*, *UNC13A*, *GRIN1*, *STX1B*, *KNCQ2*, and *CACNA1B*), strongly suggesting that similar neuroanatomical and biological substrates underlie different clinical subtypes of monogenic dystonias and other hyperkinetic movement disorders. This finding is highly consistent with the growing appreciation that many neurogenetic disorders are characterized by genetic pleiotropy and variable expressivity (Warman Chardon *et al.*, 2015), and indicates that the current system of dystonia classification based on clinical presentation may not reflect the molecular structure of the disease.

Clinically, variable expressivity may also extend to neuropsychiatric symptoms often observed in individuals with dystonia. In support of this notion, we showed that the co-expression modules enriched for DYT genes also enriched for the heritability of several neuropsychiatric disorders, including major depressive disorder, obsessive-compulsive disorder and schizophrenia. These results reinforce the concept that neuropsychiatric disorders commonly observed in dystonic patients, such as major depressive disorder and obsessive-compulsive disorder, are intrinsic to the neurobiology of dystonia. More specifically, these findings suggest that psychiatric symptoms are not merely a reaction to the disability arising from dystonia, but rather, the underlying molecular pathophysiology of dystonia increases a patient’s risk of developing psychiatric symptoms. At the same time, dysfunction of overlapping molecular pathways in the striatum may underlie the growing appreciation of movement disorders, including dystonia and other hyperkinesias, affecting drug-naïve schizophrenic patients (Peralta *et al.*, 2010; Walther and Strik, 2012; Compton *et al.*, 2015).

Notably, integration of GWAS-identified risk variants for obsessive-compulsive disorder and schizophrenia together with recent transcriptomic analyses have implicated MSNs in the neurobiology of these neuropsychiatric diseases (Skene *et al.*, 2018; Yilmaz *et al.*, 2018). We too observed an enrichment of obsessive-compulsive disorder, major depressive disorder and schizophrenia heritability in the MSN-related putamen modules, suggesting that dysfunction of MSN synaptic activity, resulting from different types of genetic insult, may represent an overlap in the biology of dystonia and these neuropsychiatric conditions. Glutamatergic pyramidal neurons have also been shown to be central to the aetiology of schizophrenia, and the ‘blue’ white-matter module, which enriched for markers of pyramidal neurons, also enriched for DYT genes and schizophrenia heritability (Skene *et al.*, 2018). Similarly, the enrichment of major depressive disorder heritability observed in the ‘blue’ white matter and ‘lightyellow’ frontal cortex modules suggests that synaptic dysfunction induced by dysregulation of dystonia genes and their interactors in glutamatergic pyramidal neurons might also underpin the high risk and occurrence of depression in dystonia patients. Altogether, these findings appear to support the concept that the same genetic disruption could operate across multiple brain regions and produce different clinical effects (i.e. dystonia versus predisposition to psychiatric symptoms), depending on the tissue-specific gene-gene interactions present.

Finally, we extended our analysis to Parkinson’s disease, a movement disorder primarily caused by dysfunction of nigral dopaminergic neurons and with significant clinical overlap with dystonia (Shetty *et al.*, 2019). We observed nominal enrichment of Parkinson’s disease heritability in the UKBEC substantia nigra ‘cyan’ module , also enriched for DOPA-responsive DYT genes, potentially suggesting an overlap that goes beyond clinical features and extends to molecular mechanisms. In support of this finding, rare and common variants in *GCH1*, the most common genetic cause of DOPA-responsive dystonia, have also been shown to increase the risk of Parkinson’s disease (Mencacci *et al.*, 2014; Nalls *et al.*, 2019). We also noted that these modules contained *SNCA*, the gene encoding alpha-synuclein. This observation may provide novel insights into the relationship between Parkinson’s disease, dystonia and dopamine metabolism, especially considering the emerging role of alpha-synuclein in synaptic biology and neurotransmitter release (Sulzer and Edwards, 2019).

### Limitations

While our analysis highlights the contribution of certain brain regions, cell types and biological functions in dystonia pathogenesis, we recognize its limitations. First and foremost, this study depends on the quality and completeness of the genetic data we use. As we can only analyse known DYT genes to identify cell types and co-expression modules of interest, our findings could change with the identification of additional disease-associated genes. This limitation might explain our failure to detect an enrichment of DYT genes in other dystonia-associated brain regions (e.g. cerebellum). Similarly, limitations in GWAS power (in particular, for anxiety and obsessive-compulsive disorder) limit our ability to assess enrichments in heritability within specific gene sets. Second, this study is limited by the availability of high quality, region and cell-specific gene expression data. Because of its completeness, we use mouse-derived cellular profiles to assess cell specificity of gene expression of the DYT genes. While we were able to replicate our enrichment of DYT genes in human dopaminergic neurons, we appreciate there may still be species differences. In addition, the co-expression networks analysed are generated through the analysis of a set of adult-derived brain regions, limiting the spatial resolution, and more importantly, preventing the analysis of potentially critical developmental windows. Hence, we may be unable to detect disease-relevant genetic interactions of DYT genes that are likely to generate dystonia through a fundamental role during brain development, such as *TOR1A*, *THAP1* and *KMT2B* (Vasudevan *et al.*, 2006; Zhao *et al.*, 2013; Faundes *et al.*, 2018). Further, this could represent one of the reasons why we could not replicate previous observations implicating *TOR1A* and *THAP1* in dopaminergic signalling (Balcioglu *et al.*, 2007; Carbon *et al.*, 2009; Napolitano *et al.*, 2010; Frederick *et al.*, 2019). Finally, given that we do not have access to significant quantities of brain transcriptomic data from individuals with genetic forms of dystonia, we assume that DYT genes operate through a loss of their normal function rather than through novel gains of function.

## Conclusion

In summary, this work enabled the unbiased identification of brain region-specific modules of biologically related genes and provided insights on cell-specific molecular signatures relevant to dystonia. We find that multiple DYT genes are functionally related in the adult human brain and likely contribute to modulation of synaptic signalling in striatal MSNs, adult dopaminergic neurons and frontal cortical neurons. While the exact mechanism of each individual DYT gene’s participation in this physiological process remains unknown, these results demonstrate a functional convergence of DYT genes linked to different phenotypic presentations and apparently unrelated cellular processes. These results bear significance for the treatment of dystonia, as future therapeutic approaches may target shared pathophysiological abnormalities, as opposed to symptoms. Finally, we demonstrate a genetic relationship between dystonia and several neuropsychiatric disorders, suggesting that disruption of genetic networks linked to dystonia pathogenesis in discrete brain regions may represent the neurobiological basis for the phenotypic overlap between dystonia and neuropsychiatric disorders.

## Funding

N.E.M. is supported by a Parkinson’s foundation grant. R.H.R. was supported through the award of a Leonard Wolfson Doctoral Training Fellowship in Neurodegeneration. This study was partially supported by Spanish grant ‘Ayudas a los Grupos y Unidades de Excelencia Científica de la Región de Murcia’, Fundación Séneca-Agencia de Ciencia y Tecnología de la Región de Murcia (19893/GERM/15, Programa de Apoyo a la Investigación 2014 and 20866/PI/18). V.V., C.W., and J.H. were supported by the UK Dementia Research Institute funded by the UK Medical Research Council (MRC), Alzheimer’s Society and Alzheimer’s Research UK. J.H. was also separately supported through the MRC. M.R. was supported by the MRC through the award of a Tenure-track Clinician Scientist Fellowship (MR/N008324/1).

## Competing interests

M.E.W. is an employee of Genomics plc, a genomics-based healthcare company. His involvement in the conduct of this research was solely in his former capacity as a Reader in Statistical Genetics at King’s College London. All other authors report no competing interests.

## Supplementary material

Supplementary material is available at *Brain* online.

## Supplementary Material

awaa217_Supplementary_Data

## Appendix 1

For full details of the UKBEC and IPDGC consortium members, see the Supplementary material.

### UK Brain Expression Consortium (UKBEC) members and affiliations

Juan A Botía, Karishma D'Sa, Paola Forabosco, Sebastian Guelfi, John Hardy, Jana Vandrovcova, Chris-Ann Mackenzie, Adaikalavan Ramasamy, Mina Ryten, Colin Smith, Daniah Trabzuni, Michael E. Weale.

### IPDGC consortium members and affiliations

UK: Alastair J. Noyce, Rauan Kaiyrzhanov, Ben Middlehurst, Demis A. Kia, Manuela Tan, Henry Houlden, Huw R. Morris, Helene Plun-Favreau, Peter Holmans, John Hardy, Daniah Trabzuni, Jose Bras, John Quinn, Kin Y. Mok, Kerri J. Kinghorn, Kimberley Billingsley, Nicholas W. Wood, Patrick Lewis, Rita Guerreiro, Ruth Lovering, Lea R’Bibo, Claudia Manzoni, Mie Rizig, Mina Ryten, Sebastian Guelfi, Valentina Escott-Price, Viorica Chelban, Thomas Foltynie, Nigel Williams, Chingiz Shashakin, Nazira Zharkinbekova, Elena Zholdybayeva, Akbota Aitkulova, Kirsten Harvey.

France: Alexis Brice, Fabrice Danjou, Suzanne Lesage, Jean-Christophe Corvol, Maria Martinez.

Germany: Anamika Giri, Claudia Schulte, Kathrin Brockmann, Javier Simón-Sánchez, Peter Heutink, Patrizia Rizzu, Manu Sharma, Thomas Gasser.

USA: Aude Nicolas, Mark R Cookson, Sara Bandres-Ciga, Cornelis Blauwendraat, David W. Craig, Faraz Faghri, J Raphael Gibbs, Dena G. Hernandez, Kendall Van Keuren-Jensen, Joshua M. Shulman, Hampton L. Leonard, Mike A. Nalls, Laurie Robak, Steven Lubbe, Steven Finkbeiner, Niccolo E. Mencacci, Codrin Lungu, Andrew B. Singleton, Sonja W. Scholz, Xylena Reed, Kendall Van Keuren-Jensen.

Canada: Ziv Gan-Or, Guy A. Rouleau.

The Netherlands: Jacobus J van Hilten, Johan Marinus.

Spain: Astrid D. Adarmes-Gómez, Miquel Aguilar, Ignacio Alvarez, Victoria Alvarez, Francisco Javier Barrero, Jesús Alberto Bergareche Yarza, Inmaculada Bernal-Bernal, Marta Blazquez, Marta Bonilla-Toribio, Juan A. Botía, María Teresa Boungiorno, Dolores Buiza-Rueda, Ana Cámara, Maria Carcel, Fátima Carrillo, Mario Carrión-Claro, Debora Cerdan, Jordi Clarimón, Yaroslau Compta, Monica Diez-Fairen, Oriol Dols-Icardo, Jacinto Duarte, Raquel Duran, Francisco Escamilla-Sevilla, Mario Ezquerra, Manel Fernández, Rubén Fernández-Santiago, Ciara Garcia, Pedro García-Ruiz, Pilar Gómez-Garre, Maria Jose Gomez Heredia, Isabel Gonzalez-Aramburu, Ana Gorostidi Pagola, Janet Hoenicka, Jon Infante, Silvia Jesús, Adriano Jimenez-Escrig, Jaime Kulisevsky, Miguel A. Labrador-Espinosa, Jose Luis Lopez-Sendon, Adolfo López de Munain Arregui, Daniel Macias, Juan Marín, Maria Jose Marti, Juan Carlos Martínez-Castrillo, Carlota Méndez-del-Barrio, Manuel Menéndez González, Adolfo Mínguez, Pablo Mir, Elisabet Mondragon Rezola, Esteban Muñoz, Javier Pagonabarraga, Pau Pastor, Francisco Perez Errazquin, Teresa Periñán-Tocino, Javier Ruiz-Martínez, Clara Ruz, Antonio Sanchez Rodriguez, María Sierra, Esther Suarez-Sanmartin, Cesar Tabernero, Juan Pablo Tartari, Cristina Tejera-Parrado, Eduard Tolosa, Francesc Valldeoriola, Laura Vargas-González, Lydia Vela, Francisco Vives.

Austria: Alexander Zimprich.

Norway: Lasse Pihlstrom.

Estonia: Pille Taba.

Australia: Sulev Koks.

## Abbreviations

EWCE =: expression-weighted cell-type enrichment;
GTEx =: Genotype-Tissue Expression Consortium;
LDSC =: linkage disequilibrium score regression;
MSN =: medium spiny neuron;
UKBEC =: UK Brain Expression Consortium;
WGCNA =: weighted gene co-expression network analysis
