# Erratum to: Dystonia genes functionally converge in specific neurons and share neurobiology with psychiatric disorders

**DOI:** 10.1093/brain/awaa366

**Published:** 2020-12-01

Niccolò E. Mencacci, Regina Reynolds, Sonia Garcia Ruiz, Jana Vandrovcova, Paola Forabosco, Alvaro Sánchez-Ferrer, Viola Volpato, UK Brain Expression Consortium, International Parkinson’s Disease Genomics Consortium, Michael E. Weale, Kailash P. Bhatia, Caleb Webber, John Hardy, Juan A. Botía, Mina Ryten. Dystonia genes functionally converge in specific neurons and share neurobiology with psychiatric disorders. Brain 2020; 143: 2771–2787. doi:10.1093/brain/awaa217.

The publisher apologizes for erroneously publishing an incorrect version of this manuscript. This has been corrected.

