# Supplementary material for: Dystonia genes functionally converge in specific neurons and share neurobiology with psychiatric disorders
Source: Brain. 2020 Aug 21;143(9):2771–87. doi: 10.1093/brain/awaa217 (PMC8354373; doi:10.1093/brain/awaa217)
Supplement: awaa217_Supplementary_Data [file awaa217_supplementary_data.zip › awaa217-suppl_data/Supplementary Figures and table legends.pdf]

Description of Supplementary Data

Supplementary Tables

Supplementary Table 1. EWCE results using dystonia gene set and mouse-derived single-cell RNA-sequencing.

Supplementary Table 2. EWCE results using dystonia gene set and human-derived single-cell RNA-sequencing.

Supplementary Table 3. Enrichment p-values of dystonia genes within gene co-expression modules generated using brain transcriptomic data from the UK Brain Expression Consortium and the GTEx consortium

Supplementary Table 4. Enrichment p-values of SynGO annotations within the substantia nigra "cyan", putamen "cyan", frontal cortex "lightyellow" and white matter "blue" modules.

Supplementary Table 5. Module preservation across four UKBEC tissues containing dystonia-linked modules.

Supplementary Table 6. Results of stratified LDSC analysis across all gene co-expression modules of interest and a range of neuropsychiatric and neurodegenerative disorders

Supplementary Figures

Supplementary Figure 1. Cell-specific profiles of dystonia-associated genes.

Plots of specificity values for all dystonia-associated genes within level 1 cell types from the Karolinska single-cell RNA-sequencing superset. Specificity values were derived from Skene et al.[2] who calculated specificity by dividing the mean expression of a gene in one cell type by the mean expression in all cell types. In other words, specificity is the proportion of a gene's total expression attributable to one cell type, with a value of 0 meaning a gene is not expressed in that cell type and a value of 1 meaning that a gene is only expressed in that cell type. For each gene, the top 3 cell types, as ranked by specificity, are marked with black borders.

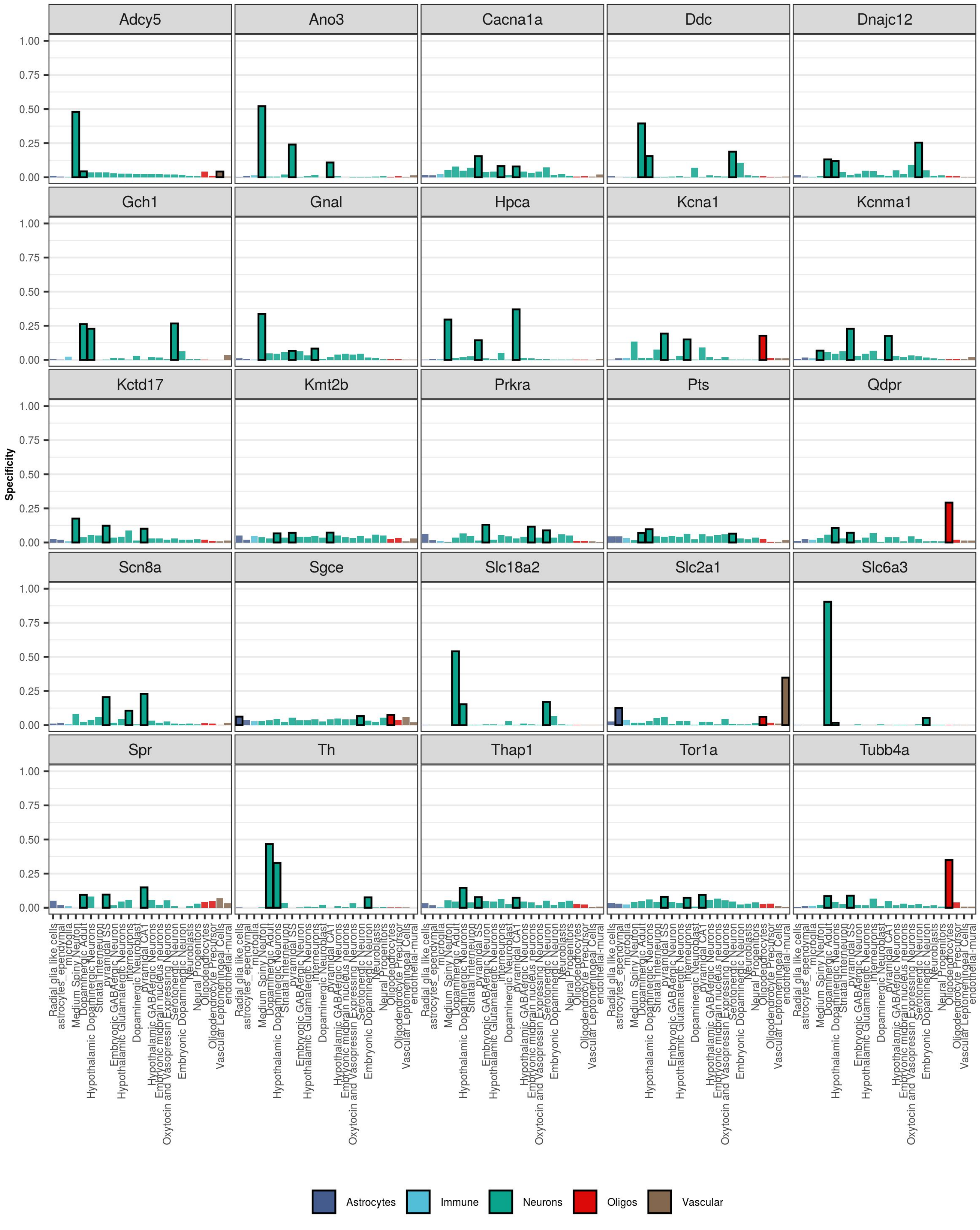
Adcy5
Ano3
Cacna1a
Ddc
Dnajc12
Gch1
Gnal
Hpca
Kcna1
Kcnma1
Kctd17
Kmt2b
Prkra
Pts
Qdpr
Scn8a
Sgce
Slc18a2
Slc2a1
Slc6a3
Spr
Th
Thap1
Tor1a
Tubb4a
Specificity
0.00
0.25
0.50
0.75
1.00
Radial glia like cells
astrocytes_ependymal
microglia
Medium Spiny Neuron
Dopaminergic Adult
Hypothalamic Dopaminergic Neurons
Striatal Interneuron
pyramidal SS
Embryonic GABAergic Neuron
Hypothalamic Glutamatergic Neurons
Interneurons
Dopaminergic Neuroblast
pyramidal CA1
Hypothalamic GABAergic Neurons
Embryonic midbrain nucleus neurons
Oxytocin and Vasopressin Expressing Neurons
Serotonergic Neuron
Embryonic Dopaminergic Neuron
Neuroblasts
Neural Progenitors
Oligodendrocytes
Oligodendrocyte Precursor
Vascular Leptomeningeal Cells
endothelial-mural
Astrocytes
Immune
Neurons
Oligos
Vascular

Supplementary Figure 2. Correlation of dystonia-associated gene specificity in murine dopaminergic and medium spiny neurons and human dopaminergic neurons.

Pairwise correlation matrix displaying: (i) in the lower triangle, scatterplots of specificity (using only dystonia-associated genes) in mouse and human cell types enriched for dystonia-associated genes; (ii) in the upper triangle, Spearman's rho for each pairwise correlation and the significance of the correlation (*, $p < 0.05$; **, $p < 0.01$; ***, $p < 0.001$); and (iii) in the diagonal, a density plot of specificity values within each cell type.

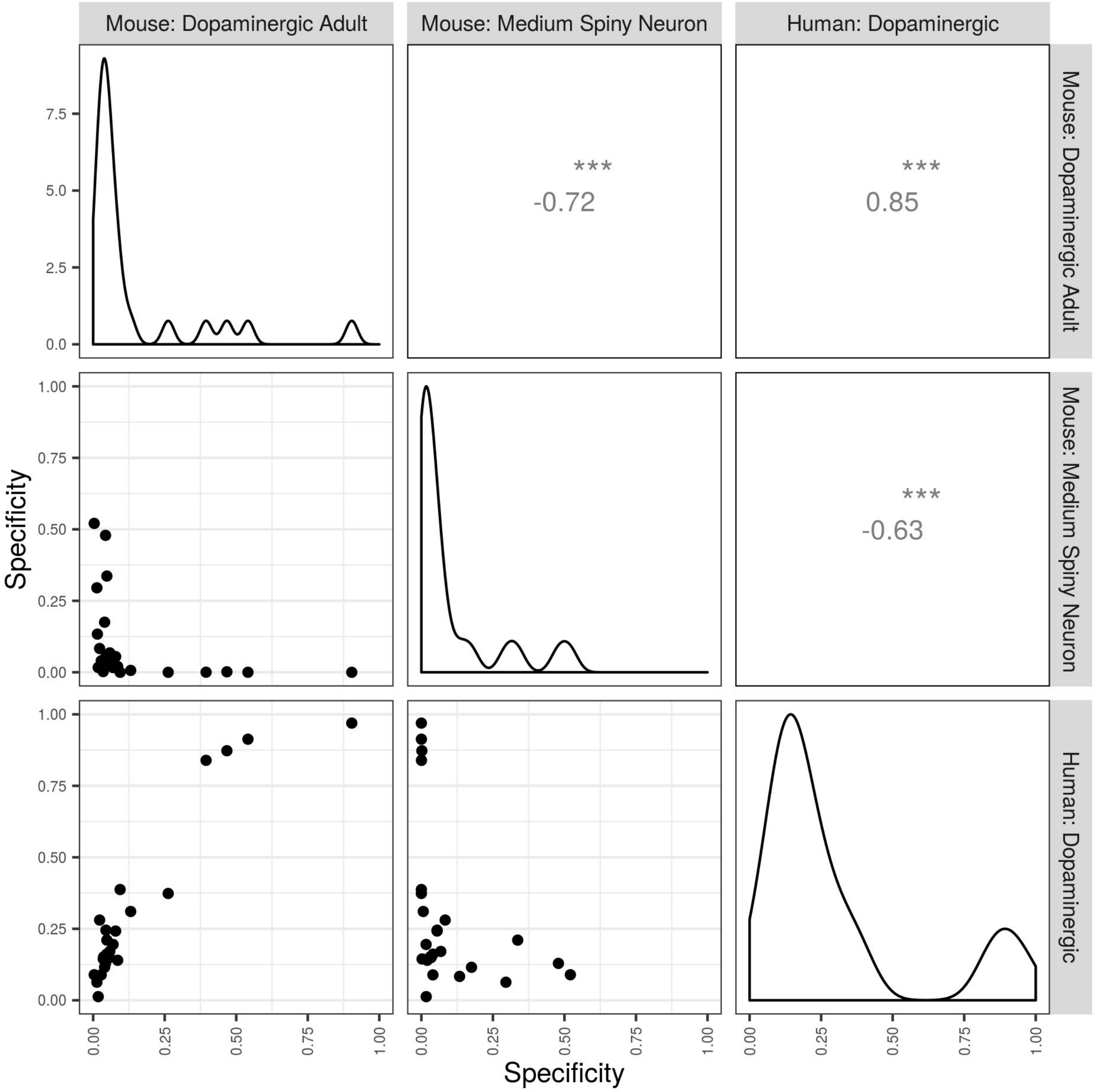

Mouse: Dopaminergic Adult
Mouse: Medium Spiny Neuron
Human: Dopaminergic
***
-0.72
***
0.85
***
-0.63
Specificity
Specificity

Supplementary Figure 3. Enrichment of disease heritability within dystonia-linked GTEx co-expression modules. Stratified LDSC using GTEx co-expression modules. The black dashed lines indicate the cut-off for Bonferroni significance ($p < 0.05/(3 \times 5)$). Bonferroni-significant results are marked with black borders. Numerical results are reported in **Supplementary Table 6**. FCTX, frontal cortex; PUTM, putamen; MDD, major depressive disorder; OCD, obsessive compulsive disorder; PD, Parkinson's disease; SCZ, schizophrenia; SNIG, substantia nigra.

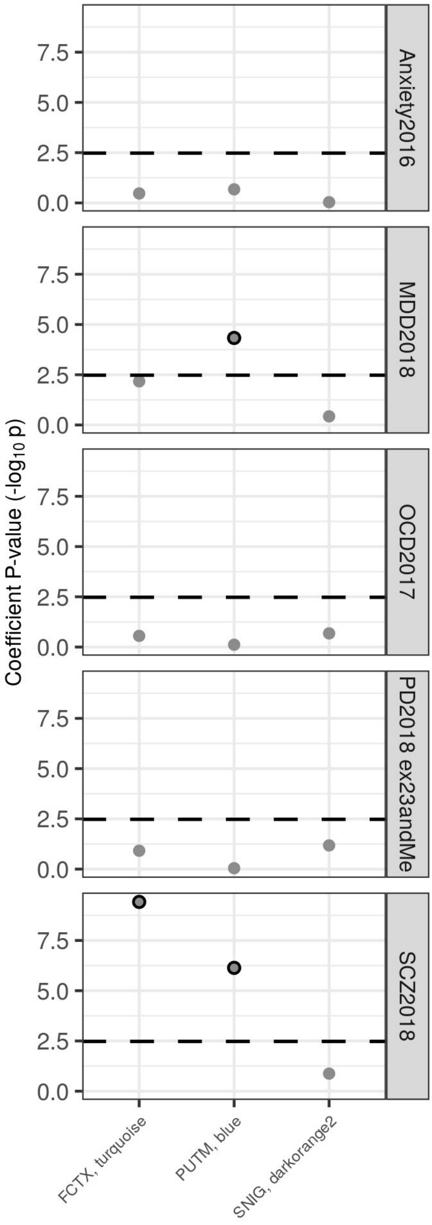

Coefficient P-value (-log10 p)
Anxiety2016
MDD2018
OCD2017
PD2018 ex23andMe
SCZ2018
0.0
2.5
5.0
7.5
FCTX, turquoise
PUTM, blue
SNIG, darkorange2
